# Comparative Effects of Rosuvastatin and Atorvastatin Loading Doses on Immediate Post-percutaneous Coronary Intervention (PCI) Thrombolysis in Myocardial Infarction (TIMI) Flow in ST-Segment Elevation Myocardial Infarction (STEMI) Patients

**DOI:** 10.7759/cureus.74076

**Published:** 2024-11-20

**Authors:** Ahmed Jamal Chaudhary, Sana Iqbal, Nisar Ahmad Khan, Anshaal Furrukh, Shah Bano, Muhammad Nasir Ali, Afaq Ali, Sajjad Ullah Khan

**Affiliations:** 1 Medicine / Transitional Medicine, Detroit Medical Center (DMC) Sinai-Grace Hospital, Detroit, USA; 2 Medicine, Lahore Medical and Dental College, Lahore, PAK; 3 Internal Medicine, Detroit Medical Center (DMC) Sinai-Grace Hospital, Detroit, USA; 4 Medicine Department, St. Luke's Hospital, Kilkenny, IRL; 5 Medicine and Surgery, Jinnah Medical and Dental College, Karachi, PAK; 6 Medicine and Surgery, Shalamar Medical and Dental College, Lahore, PAK; 7 Medicine Department, Abbasi Shaheed Hospital, Karachi, PAK; 8 Medicine and Surgery, Rai Medical College, Sargodha, PAK; 9 Interventional Cardiology Department, Lady Reading Hospital, Peshawar, PAK

**Keywords:** atorvastatin, cardiovascular outcomes, myocardial reperfusion, pci, rosuvastatin, stemi

## Abstract

Background: Primary percutaneous coronary intervention (PCI) is crucial in managing acute ST-segment elevation myocardial infarction (STEMI), emphasizing the importance of optimal myocardial reperfusion.

Objective: The goal of this research was to determine how loading doses of rosuvastatin and atorvastatin affected the flow rate of thrombolysis in myocardial infarction (TIMI) immediately post-perfusion thrombolysis in patients undergoing primary PCI.

Methodology: This prospective, comparative study was carried out over a one-year period (January 2023 to December 2023) in Pakistan. Data was gathered from patient interviews and electronic medical records for adult patients receiving primary PCI. Interventional cardiologists who were blinded to the evaluation of the immediate post-perfusion TIMI flow conducted statistical analysis to compare the results between the two statin groups.

Results: Both groups had good procedural success rates: 184 patients (92.00%) in the group using rosuvastatin and 179 patients (89.50%) in the group on atorvastatin (p = 0.284). A comparable use of auxiliary equipment was seen, with 103 patients (51.50%) and 97 patients (48.50%) in the atorvastatin group and 108 patients (54.00%) and 92 patients (46.00%) in the rosuvastatin group, respectively (p = 0.53). There were no notable variations in the immediate post-perfusion TIMI flow grades either, with p-values of 0.532 for normal flow and 0.421 for no-reflow. The two groups' mean lengths of hospital stays were comparable, measuring 3.5 days (± 1.2) for the rosuvastatin group and 3.8 days (± 1.3) for the atorvastatin group (p = 0.321).

Conclusion: Rosuvastatin and atorvastatin had comparable rapid post-perfusion TIMI flows in the initial PCI participants, indicating that they may be used interchangeably to maximize myocardial reperfusion in acute STEMI.

## Introduction

Acute ST-segment elevation myocardial infarction (STEMI) remains a significant contributor to cardiovascular morbidity and mortality worldwide, with its prevalence continuing to rise due to lifestyle factors and an aging population [1,2]. Primary percutaneous coronary intervention (PCI) is the gold standard for managing STEMI, aimed at rapidly restoring coronary artery patency to salvage ischemic myocardium and reduce the risk of adverse cardiac events such as heart failure or recurrent myocardial infarction [3]. Despite advancements in procedural techniques and adjunct pharmacotherapy, achieving optimal myocardial reperfusion remains essential to improving clinical outcomes and reducing STEMI-related mortality [4].

The thrombolysis in myocardial infarction (TIMI) flow grade is a critical indicator of coronary reperfusion after PCI, offering a standardized measure to assess the restoration of blood flow in the infarct-related artery [5]. Higher TIMI flow grades post-PCI are correlated with better patient prognoses, including improved left ventricular function, reduced adverse cardiac events, and enhanced long-term survival rates [6,7]. Thus, optimizing TIMI flow during PCI is crucial to ensuring favorable outcomes in patients with acute STEMI [8].

Statin therapy plays a vital role in the secondary prevention of cardiovascular events due to its serum cholesterol level-lowering and pleiotropic effects, such as anti-inflammatory, antioxidant, and endothelial-stabilizing properties [9,10]. These additional effects of statins may further enhance myocardial reperfusion, making them a critical component of the therapeutic regimen in STEMI management [11]. Among the statins, atorvastatin and rosuvastatin are frequently used due to their proven efficacy in reducing cardiovascular morbidity and mortality [12].

However, while the safety and serum cholesterol level-lowering capabilities of both rosuvastatin and atorvastatin are well-documented, there remains limited data on their comparative effects on immediate post-perfusion TIMI flow in patients undergoing primary PCI [13]. Understanding these differences is clinically relevant, as optimizing the loading dose of statins before PCI could significantly influence myocardial reperfusion and subsequent patient outcomes. Given the increasing incidence of STEMI, refining pharmacotherapy to enhance reperfusion strategies is of the utmost importance.

This study seeks to bridge the existing knowledge gap by directly comparing the effects of loading doses of rosuvastatin versus atorvastatin on immediate post-perfusion TIMI flow in STEMI patients undergoing primary PCI. By evaluating whether one statin offers superior benefits in this critical setting, the research aims to optimize acute management strategies, enhance reperfusion outcomes, and potentially reduce the burden of cardiovascular disease. The evidence generated could guide pharmacological decisions to improve patient outcomes in the management of acute STEMI.

## Materials and methods

Study design and settings

The Abbasi Shaheed Hospital (Karachi) and Lady Reading Hospital (Peshawar), tertiary care facilities for cardiovascular treatment in Pakistan, were the sites of this research. The patients who presented with acute STEMI and had primary PCI were included in the trial, which ran from January 2023 to December 2023.

Inclusion and exclusion criteria

Adult patients (either gender) who received primary PCI throughout the length of the trial and who presented with acute STEMI were included in the study. Exclusions from the study included patients with renal impairment (estimated glomerular filtration rate < 30 ml/minute/1.73 m^2^), liver dysfunction (alanine transaminase or aspartate transaminase levels more than three times the upper limit of normal), history of statin intolerance or allergic reactions, and contraindications to statin therapy.

Sample size

A power analysis with an impact size of 0.5, a power of 80%, and a statistical significance level of 0.05 served as the basis for calculating the sample size. To identify statistically significant changes in immediate post-perfusion TIMI flow between the two research groups, a minimum sample size of 400 patients was required.

Data collection

Clinical information was gathered prospectively from electronic medical records and patient interviews. This information included demographics, medical history, test results, and procedure specifics. By using angiography, skilled interventional cardiologists blinded to the patients' statin assignment evaluated the immediate post-perfusion TIMI flow. Standardized data collection forms were used to guarantee accuracy and uniformity in the data-gathering process.

Dosage administration

Statins, such as atorvastatin and rosuvastatin, were administered according to established procedures that were customized for each patient. When necessary, rosuvastatin was recommended at a loading dose of 10 mg for quick therapeutic benefits in addition to the usual daily dosage of 20 mg. In a comparable manner, 40 mg of atorvastatin was first prescribed daily, with a loading dose of 20 mg added for any required fast-acting effects.

Statistical analysis

Descriptive statistics were used to describe the operational parameters and baseline features. Frequencies and percentages were used to describe data that is categorical, while mean ± standard deviation or median with interquartile ranges were used to display variables that are continuous. Between-group analyses were carried out using appropriate statistical methods, such as chi-square tests for categorical data and independent t-tests for continuous variables. P-values were considered highly significant if they were less than 0.05.

Ethical approval

The study received ethical approval from the Institutional Review Boards (IRBs) of Lahore Medical and Dental College, Lahore, Pakistan, Abbasi Shaheed Hospital, Karachi, Pakistan, and Lady Reading Hospital, Peshawar, Pakistan.

## Results

The baseline characteristics of patients receiving primary PCI in the atorvastatin and rosuvastatin groups are shown in Table 1. The average age of the rosuvastatin group was 62.4 (± 8.3) years, with 118 patients (59.00%) under 65 and 82 patients (41.00%) over 65. The distribution of patients' genders revealed that 151 patients (75.50%) were males, while 49 of the patients (24.50%) were females. Of the comorbidities, 116 patients (58.00%) had hypertension, 83 (41.50%) had diabetes mellitus, and 42 (21.00%) had a history of smoking. Of the patients, 93 (46.50%) had a history of dyslipidemia, 54 (27.00%) had a history of myocardial infarction (MI), 27 (13.50%) had a history of PCI, and 16 (8.00%) had a history of coronary artery bypass grafting (CABG). The mean age of the atorvastatin group was 63.1 years (± 7.9), with 84 patients (42.00%) and 116 patients (58.00%) being younger than 65. There were 46 girls (23.00%) and 154 men (77.00%) in the gender distribution. Comorbidities comprised diabetes mellitus in 92 individuals (46.0%), hypertension in 109 people (54.50%), and a past of smoking in 36 individuals (18.00%). Dyslipidemia was noted in 98 patients (49.00%), MI in 48 patients (24.0%), PCI in 33 patients (16.50%), and CABG in 21 patients (10.50%).

**Table 1:** Patient overview and initial features PCI: percutaneous coronary intervention; CABG: coronary artery bypass grafting

Characteristic		Rosuvastatin group	Atorvastatin group
Age (years)	Mean ± SD	62.4 ± 8.3	63.1 ± 7.9
Age group categories n (%)	< 65 years	118 (59.00%)	116 (58.00%)
	≥ 65 years	82 (41.00%)	84 (42.00%)
Gender categories n (%)	Male	151 (75.50%)	154 (77.00%)
	Female	49 (24.50%)	46 (23.00%)
Comorbidities n (%)	Hypertension	116 (58.00%)	109 (54.50%)
	Diabetes mellitus	83 (41.50%)	92 (46.00%)
	Smoking	42 (21.00%)	36 (18.00%)
	History of dyslipidemia	93 (46.50%)	98 (49.00%)
	Previous myocardial infarction	54 (27.00%)	48 (24.00%)
	Previous PCI	27 (13.50%)	33 (16.50%)
	Previous CABG	16 (8.00%)	21 (10.50%)

Procedure information for primary PCI patients is shown in Table 2, which is divided into groups based on statin therapy. According to the American College of Cardiology and the American Heart Association (ACC/AHA) criteria, 139 patients (69.50%) in the rosuvastatin group and 131 patients (65.50%) in the atorvastatin group had type A lesions (p = 0.421). With 184 patients (92.00%) in the rosuvastatin group and 179 patients (89.50%) in the atorvastatin group (p = 0.284), both groups had good procedural success rates. Similarities were also seen in the use of stents and angioplasty balloons among the dual groups: 108 patients (54.00%) and 92 patients (46.00%) in the group taking rosuvastatin, and 103 patients (51.50%) and 97 patients (48.50%) in the group using atorvastatin, respectively (p = 0.53).

**Table 2:** Achievement rates and procedural features ACC/AHA: American College of Cardiology/American Heart Association

Procedural details		Rosuvastatin group		Atorvastatin group		p-value
		n	%	n	%	
Lesion complexity (ACC/AHA)	Type A	139	69.50	131	65.50	0.421
	Type B	61	30.50	69	34.50	
Procedural success	Successful	184	92.00	179	89.50	0.284
	Unsuccessful	16	8.00	21	10.50	
Adjunctive devices used	Angioplasty balloon	108	54.00	103	51.50	0.53
	Stent	92	46.00	97	48.50	

The TIMI flow grades immediately after perfusion in individuals treated with atorvastatin or rosuvastatin are shown in Table 3. Grade 0, which indicates no perfusion; grade 1, which indicates penetration without perfusion; Grade 2, which indicates partial perfusion; and normal flow are the four categories into which the TIMI flow grades are divided. Six patients (3.00%) had grade 1, five patients (2.50%) had grade 2, and 184 patients (92.00%) had normal flow in the rosuvastatin group. Five patients (2.50%) had grade 0. The atorvastatin group, in contrast, consisted of 179 patients (89.50%) with normal flow, eight patients (4.00%) with grade 1, seven patients (3.50%) with grade 2, and six patients (3.00%) with grade 0. The p-values, which are 0.421 for no-reflow and 0.532 for normal flow, show the statistical significance of the differences between the two groups.

**Table 3:** TIMI flow grades following instantaneous perfusion TIMI: thrombolysis in myocardial infarction

TIMI flow grade		Rosuvastatin group		Atorvastatin group		p-value
		n	%	n	%	
No-reflow	Grade 0: No perfusion	5	2.50	6	3.00	0.421
	Grade 1: Penetration without perfusion	6	3.00	8	4.00	
	Grade 2: Partial perfusion	5	2.50	7	3.50	
Normal flow		184	92.00	179	89.50	0.532

**Figure 1:**
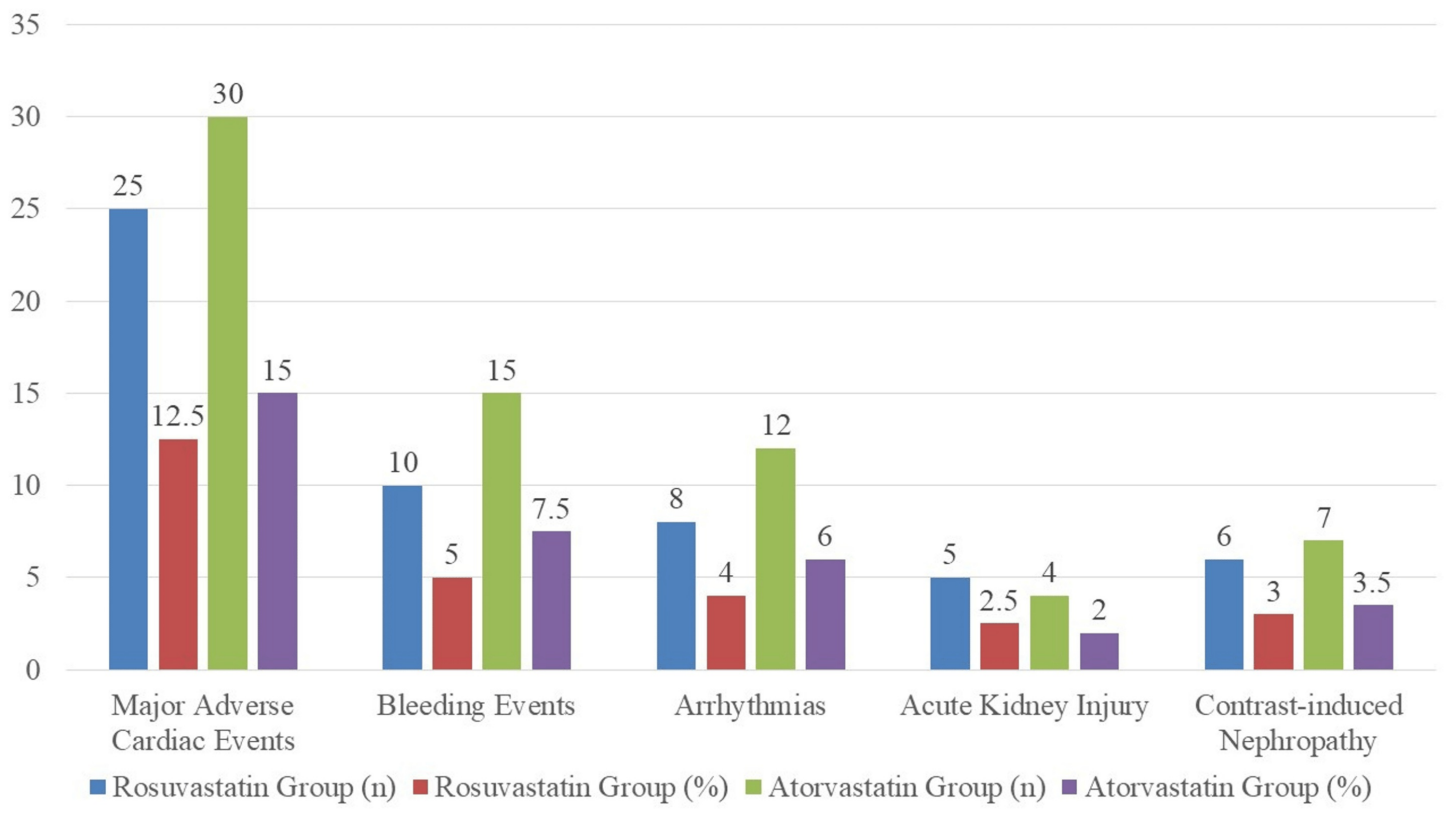
Comparing adverse events in the groups taking atorvastatin and rosuvastatin

A comparison of side effects between the atorvastatin and rosuvastatin groups is shown in Figure 1. Major adverse cardiac occurrences, bleeding events, arrhythmias, acute kidney injury, and contrast-induced nephropathy are examples of adverse occurrences. Among the patients in the rosuvastatin group, there were 25 (12.50%) who had major adverse cardiac events, 10 (5.00%) who had bleeding events, eight (4.00%) who had arrhythmias, five (2.50%) who had acute kidney injury, and six (3.00%) who acquired contrast-induced nephropathy. In contrast, 30 patients (15.00%) in the atorvastatin group experienced major adverse cardiac events, 15 patients (7.50%) had bleeding events, 12 patients (6.00%) had arrhythmias, four patients (2.00%) had acute kidney injury, and seven patients (3.50%) developed contrast-induced nephropathy.

The comparison of several laboratory parameters between the atorvastatin and rosuvastatin groups is shown in Table 4. The low-density lipoprotein (LDL) cholesterol levels in the rosuvastatin and atorvastatin groups were 100.5 ± 15.3 mg/dL and 102.1 ± 14.8 mg/dL, respectively, with mean ± SD values. The interquartile range (IQR) for alaminase (ALT) was 25 (20-30) IU/L in the rosuvastatin group and 28 (22-32) IU/L in the atorvastatin group. The mean ± standard deviation (SD) values for the estimated glomerular filtration rate (eGFR) were 76.8 ± 11.2 ml/minute/1.73 m^2^ in the atorvastatin group and 78.2 ± 10.5 ml/minute/1.73 m^2^ in the rosuvastatin group. The levels of creatine kinase-MB (CK-MB) in the rosuvastatin and atorvastatin groups were 12.5 (10-15) ng/mL and 13 (11-16) ng/mL, respectively, with median (IQR) values. The mean ± SD values of troponin I levels were 6.8 ± 2.1 ng/mL in the group using rosuvastatin and 7.2 ± 2.5 ng/mL in the group taking atorvastatin. The mean ± standard deviation of hemoglobin levels in the rosuvastatin and atorvastatin groups were 13.8 ± 1.2 g/dL and 13.5 ± 1.3 g/dL, respectively. In conclusion, the mean ± standard deviation of platelet counts (x10^9^/L) was found to be 220.4 ± 45.6 x109/L for the rosuvastatin group and 215.8 ± 42.3 x10^9^/L for the atorvastatin group. Hemoglobin levels (p = 0.142) and ALT levels (p = 0.284) showed significant changes, but no other parameters showed any significant differences (p > 0.05).

**Table 4:** Comparison of laboratory parameters for the atorvastatin and rosuvastatin groups LDL cholesterol: low-density lipoprotein cholesterol, ALT: alanine transaminase, eGFR: estimated glomerular filtration rate, CK-MB: creatine kinase-MB

Laboratory parameter	Rosuvastatin group	Atorvastatin group	p-value
LDL cholesterol (mg/dL), mean ± SD	100.5 ± 15.3	102.1 ± 14.8	0.421
ALT (IU/L), median (IQR)	25 (20-30)	28 (22-32)	0.284
eGFR (ml/min/1.73 m2), mean ± SD	78.2 ± 10.5	76.8 ± 11.2	0.532
CK-MB (ng/mL), median (IQR)	12.5 (10-15)	13 (11-16)	0.176
Troponin I (ng/mL), mean ± SD	6.8 ± 2.1	7.2 ± 2.5	0.321
Hemoglobin (g/dL), mean ± SD	13.8 ± 1.2	13.5 ± 1.3	0.142
Platelet count (x109/L), mean ± SD	220.4 ± 45.6	215.8 ± 42.3	0.621

Table 5 displays clinical outcomes and the duration of hospital stay for the atorvastatin and rosuvastatin-taking groups. In the group using rosuvastatin, the average length of hospital stay was 3.5 days (± 1.2), whereas in the group on atorvastatin, it was 3.8 days (± 1.3). The statistical significance of the disparity in hospital stay duration among the two subgroups was not found, as shown by the comparison's p-value of 0.321.

**Table 5:** Hospital stay duration and clinical outcomes

Hospital stay duration (days)	Rosuvastatin group	Atorvastatin group	p-value
Mean ± SD	3.5 ± 1.2	3.8 ± 1.3	0.321

## Discussion

The immediate post-perfusion TIMI flow is a crucial determinant of myocardial reperfusion and clinical outcomes following primary PCI in acute STEMI patients [14]. Our study found comparable rates of achieving optimal TIMI flow between atorvastatin and rosuvastatin groups (92.00% vs. 89.50%), which aligns with previous research indicating similar efficacy in restoring coronary flow [15,16]. This suggests that both statins are effective options in the acute setting, providing clinicians with flexibility based on patient-specific factors. Additionally, procedural success rates and lesion complexity were similar across groups, reinforcing the role of statins in optimizing PCI outcomes by stabilizing plaques and enhancing coronary flow [17,18].

Regarding safety, both statins demonstrated a similar adverse event profile. The rates of serious adverse cardiac events, bleeding episodes, and renal complications did not significantly differ between the two groups, which is in agreement with the existing literature showing that atorvastatin and rosuvastatin are generally well-tolerated in high-risk cardiovascular patients [19,20]. For instance, bleeding rates were slightly lower in the rosuvastatin group (5.00% vs. 7.50%), but this difference was not statistically significant. The low incidence of adverse renal events and arrhythmias further confirms the safety of these agents when used at high doses during the acute phase of STEMI management.

Interestingly, our findings on comparable clinical outcomes align with studies that reported no substantial differences in myocardial perfusion or adverse events when comparing high-dose regimens of various statins in acute coronary syndromes [15,21]. However, some evidence suggests that rosuvastatin might have a marginal advantage due to its more potent anti-inflammatory effects, although this was not statistically significant in our cohort [22]. Future research, especially large-scale randomized trials, would be beneficial to determine if such differences might impact long-term outcomes.

Beyond the immediate pharmacological effects, social determinants of cardiovascular health, as highlighted in studies like that of Borkowski et al., play a pivotal role in patient outcomes [23]. In regions like Pakistan, where our study was conducted, factors such as socioeconomic status, limited access to timely medical care, and disparities in healthcare resources significantly influence the prognoses of STEMI patients. These systemic barriers can lead to delayed interventions, which in turn may compromise the benefits of pharmacological therapies. Addressing these social inequities, along with optimizing acute pharmacotherapy, could lead to better health outcomes.

Strength and limitation

The present study possesses several strengths, notably its prospective design and comparative approach, which significantly reduce recall bias and enhance the reliability of collected data. By directly comparing the effects of loading doses of rosuvastatin and atorvastatin on immediate post-perfusion TIMI flow, the study offers valuable insights into optimizing acute management strategies for STEMI patients undergoing primary PCI. The blinded evaluation of TIMI flow by interventional cardiologists further strengthens the study’s methodological rigor, reducing the risk of assessment bias. However, a key limitation is the lack of randomization in patient allocation, which introduces the potential for selection bias, as patients were assigned to either statin therapy based on clinical practice or physician discretion rather than a randomized process. Statistical adjustments were employed to mitigate this limitation, and blinding was utilized during the evaluation of primary outcomes.

Future perspective

Although this study provides insights into the acute effects of atorvastatin and rosuvastatin on immediate myocardial reperfusion in STEMI patients, future research should focus on assessing the long-term impact of these statins on mortality, recurrent cardiovascular events, and overall patient survival. Additionally, an analysis of specific patient subgroups-such as those defined by age, comorbidities (e.g., diabetes, hypertension), and genetic markers-could provide valuable insights into whether certain populations derive greater benefit from one statin over the other. Investigating the mechanistic differences between statins, especially their effects on endothelial function, plaque stability, and inflammation, will also help further refine treatment strategies in the acute coronary syndrome setting. A randomized controlled trial design would be essential to minimize bias and strengthen the evidence supporting the clinical use of statins in STEMI management.

## Conclusions

The results demonstrated no significant differences between atorvastatin and rosuvastatin in terms of achieving optimal TIMI flow, procedural success rates, lesion complexity, adverse events, laboratory parameters, length of hospital stay, or overall clinical outcomes. Both statins showed comparable effectiveness and safety profiles, suggesting that they may be used interchangeably in the acute STEMI setting. These findings underscore the clinical importance of statin therapy in primary PCI for acute STEMI patients, as both atorvastatin and rosuvastatin contribute to myocardial reperfusion and potentially improve patient outcomes. These results could inform clinical practice and guidelines, reinforcing the use of either statin as a valuable therapeutic option in the management of acute STEMI, thereby helping to optimize patient care and treatment strategies.
